# Supplementary material for: Production of individualized V gene databases reveals high levels of immunoglobulin genetic diversity
Source: Nat Commun. 2016 Dec 20;7:13642. doi: 10.1038/ncomms13642 (PMC5187446; doi:10.1038/ncomms13642)
Supplement: Supplementary Information — Supplementary Figures 1-3 and Supplementary Tables 1-2 [file ncomms13642-s1.pdf]

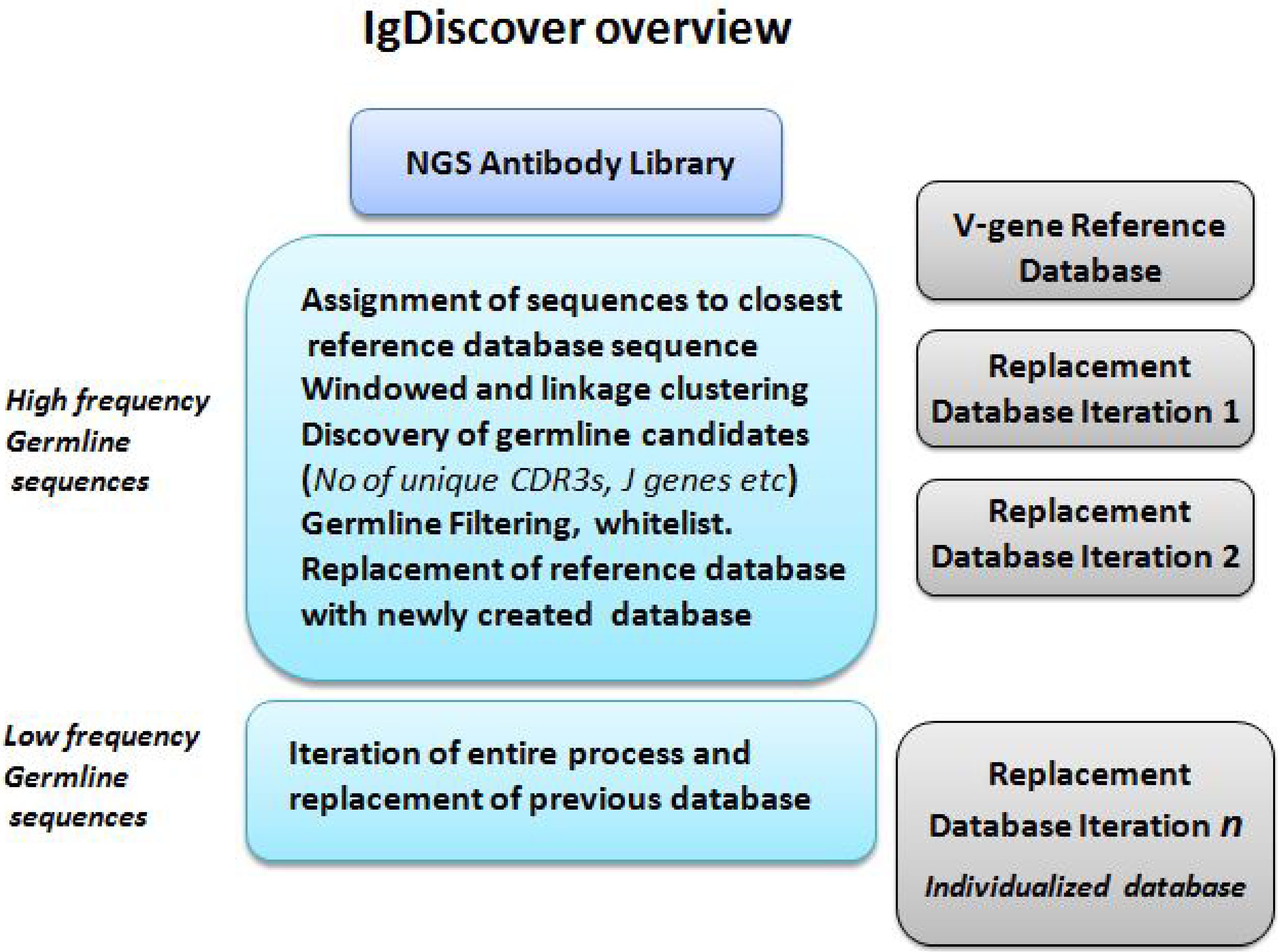

Supplementary Figure 1. Schematic overview of IgDiscover process

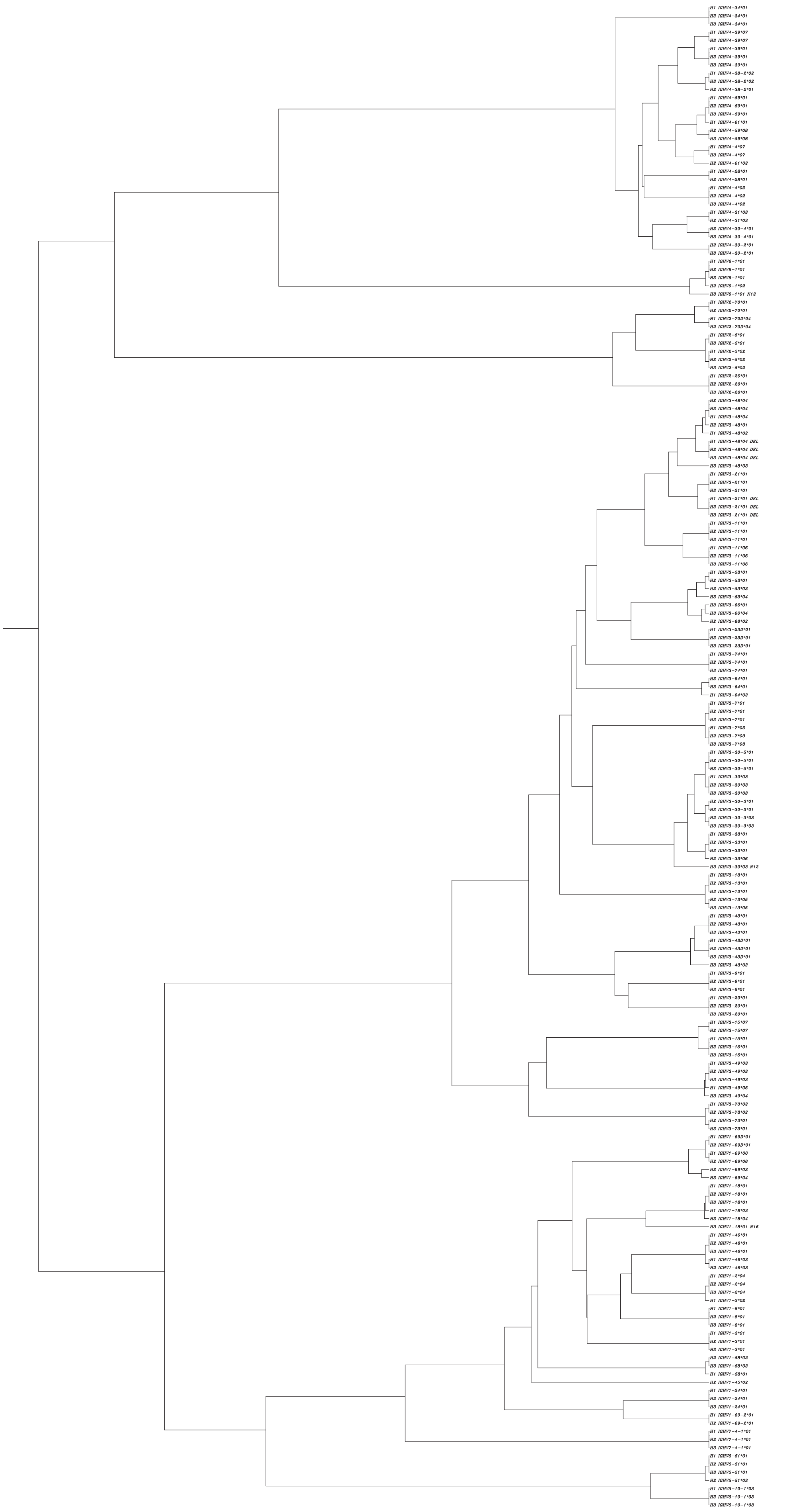

Supplementary Figure 2. Human individualized VH germline phylogenetic tree. Phylogenetic relationship between VH germline genes from the individualized V gene databases from individuals H1, H2, and H3.

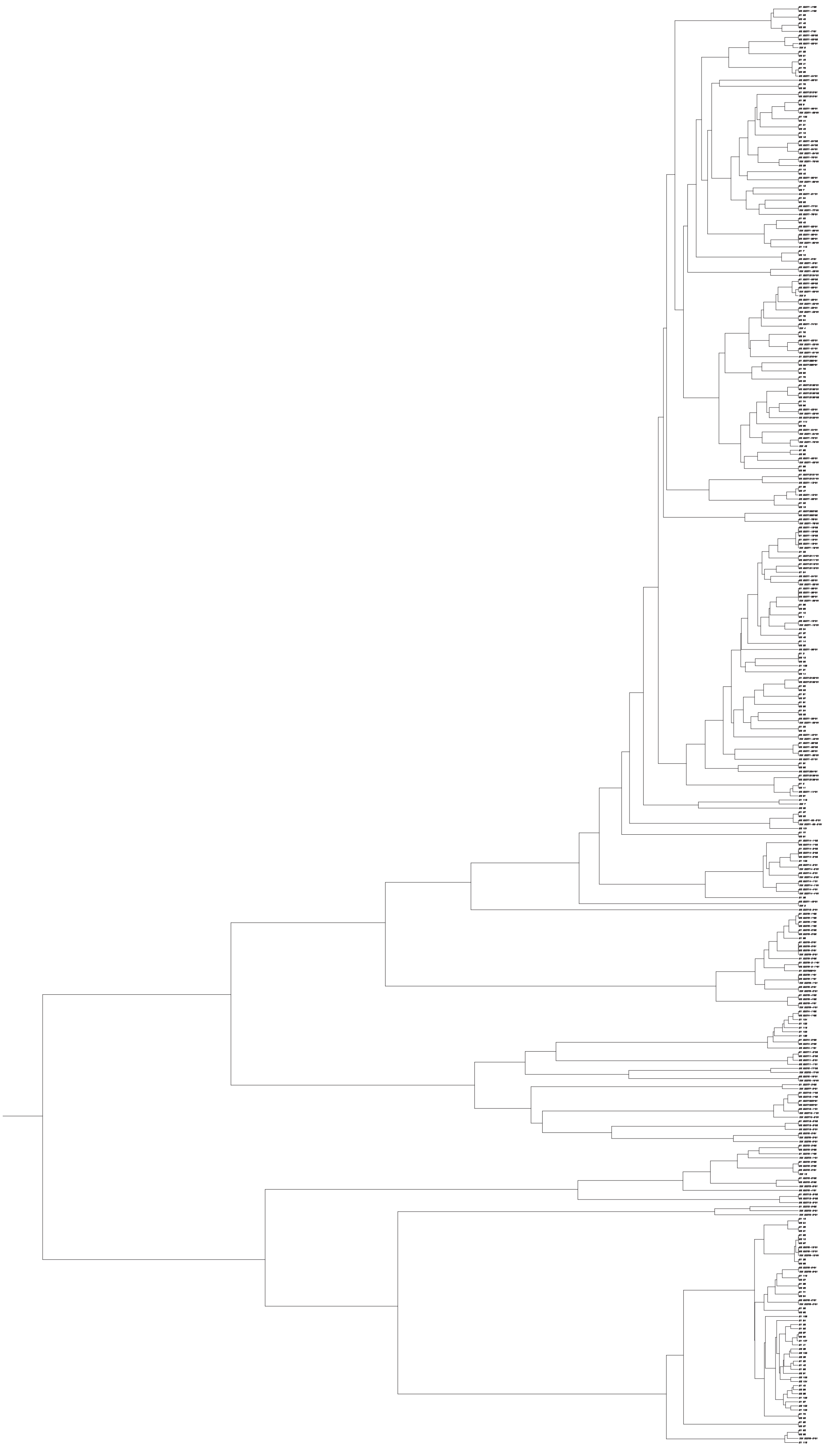

Supplementary Figure 3. Phylogenetic tree of 4 individualized mouse VH germline databases, from two BALB/c (M1 and M2) and two C57BL6 mice (M3 and ION)

Supplementary Table 1.

**Multiplex Primer**	**Sequence**
M_VH15F	CCTACACGACGCTCTTCCGATCTGGACTGGATTTGGATCACTCTCCATCTGC
M14_F	CCTACACGACGCTCTTCCGATCTCCTGATGGCAGTGGTTACAGGGGTCA
M_13F	CCTACACGACGCTCTTCCGATCTGTGGCTCTTTTGAACGGTGTCCAGTG
M_12F	CCTACACGACGCTCTTCCGATCTGGTGACAGTCCTTCCTGGTAGCCTGTC
M_11F	CCTACACGACGCTCTTCCGATCTCTCACGTCTCAACATGGAGTGGGAACTGAGCT
M_10F	CCTACACGACGCTCTTCCGATCTATGCTGTTGGGGCTGAAGTGGGTT
M_9BF	CCTACACGACGCTCTTCCGATCTGGCAGCAGCTCAAAGTATCCAAGC
M_9AF	CCTACACGACGCTCTTCCGATCTGGCAGCTGCCCAAAGTGCCCAAGC
M_8F	CCTACACGACGCTCTTCCGATCTCCTGCTGCTGATTGTCCCTGCATATGTCCTGTC
M_7F	CCTACACGACGCTCTTCCGATCTCACACATCCCTTACCATGGATTTTGGGCTGA
M_6F	CCTACACGACGCTCTTCCGATCTCACCATGGACTTGAGACTGAGCTGTGCT
M_5F	CCTACACGACGCTCTTCCGATCTCCAGTCACCATGTACTTCAGGCTCAGCTCAG
M_4F	CCTACACGACGCTCTTCCGATCTCACACATCCCTTACCATGGATTTTGGGCTGA
M_3F	CCTACACGACGCTCTTCCGATCTCCTGTTGACAGCCATTCCTGGTATCCTGT
M_2F	CCTACACGACGCTCTTCCGATCTCTCCTGTCAGGAACTGCAGGTGTCCTCT
M_1DF	CCTACACGACGCTCTTCCGATCTGGTAKCAGCAGCTACAGGTGTCCACTC
M_1CF	CCTACACGACGCTCTTCCGATCTCCTGTCAGGAACTGCAGGTGTCCATTG
M_1BF	CCTACACGACGCTCTTCCGATCTCCTGTCAGKAAYTGCAGGTGTCCAMTC
M_1AF	CCTACACGACGCTCTTCCGATCTCCTGTCAGGAACTGCAGGTGTCCAATC
Hum_7F	CCTACACGACGCTCTTCCGATCTGGTGGCAGCAGCAACAGGTGCCCACT
Hum_6F	CCTACACGACGCTCTTCCGATCTGGCCTCCCATGGGGTGTCCTGTC
Hum_5F	CCTACACGACGCTCTTCCGATCTCTGGCTGTTCTCCAAGGAGTCTGTG
Hum_4F	CCTACACGACGCTCTTCCGATCTGGTGGCRGCTCCCAGATGGGTCCTGTC
Hum_3DF	CCTACACGACGCTCTTCCGATCTGGGTTTTCCTTKTKGCTATWTTAGAAGGTGTCCAGTG
Hum_3CF	CCTACACGACGCTCTTCCGATCTGGATTTTCCTTGCTGCTATTTTAAAAGGTGTCCAGTG
Hum_3BF	CCTACACGACGCTCTTCCGATCTGGGTTTTCCTTGTTGCTATTTTAAAAGGTGTCCARTG
Hum_3AF	CCTACACGACGCTCTTCCGATCTGGGTTTTCCTCGTTGCTCTTTTAAGAGGTGTCCAGTG
Hum_2BF	CCTACACGACGCTCTTCCGATCTCCTGCTACTGACTGTCCCGTCCTGGGTCTTATC
Hum_2AF	CCTACACGACGCTCTTCCGATCTCCTGCTGCTGACCAYCCCTTCMTGGGTCTTGTC
Hum_1EF	CTACACGACGCTCTTCCGATCTGCTGGCTGTAGCTCCAGGTGCTCACTC
Hum_1DF	CCTACACGACGCTCTTCCGATCTGGTGGSAGCAGCAACARGWGCCCACTC
Hum_1CF	CCTACACGACGCTCTTCCGATCTGGTGGCAGCAGCTACAGGTGTCCAGTC
Hum_1BF	CCTACACGACGCTCTTCCGATCTGGTGGCAGCAGCCACAGGTGCCCACTC
Hum_1AF	CCTACACGACGCTCTTCCGATCTGGTGGCAGCAGTCACAGATGCCTACTC
**Race Primers**	**Sequence**
SM_RACE1	CGTGAGCTGAGTACGACTCACTATAGCTTCAC(N12)rGrGrGrGrG
F_Universal	CGTGAGCTGAGTACGACTCACTATAGCTTC
IgM_RevHuman	CGGGGAATTCTCACAGGAGACGAGGGGGAAAAG
IgM_RevRhesus	GGGGCATTCTCACAGGAGACGAGGGGGAAAAG
IgM_RevMouse	GGGGGAAGACATTTGGGAAGGACTGACTCT
IgK_RevRhesus	GGGATAGAAGTTATTCAGCAGGCACACAACAGAG
IgL_RevRhesus	CACTGATCAGACACACTAGTGTGGCCTTG

Genomic Validation Primers

ID	Forward primer	Reverse primer
VH1_23	GGCGTGGTCCACGTGTCACCTATCTTCTTCC	CCCACTCCATGAATGTTACTTACAGTG
VH1_36	CCCACAGTAGGTTCACACCCGGTAAAATCAGG	CACAGCTGCCTTCTCCCTCAGGGTTTC
VH1_53	GCCCAGAGAGCATCACACAACAACC	GGCTGCCTTTCCCACTCTGTGAATG
VH1_59	GGGTGGGGTGGCTTGAGCTATGAAATACC	CGGCTTGATTGATGGCTGCCTTTCC
VH1_61	GAGGGCAAGGCCCAGGAAAGTTCAGG	CAGCTGCCTCCTCCCTCAGGGTTTC
VH2_12	GGCACCCACAGGAAACCACCACAC	CTCCTGAGTCCTGAGACCTGAGTGCAC
VH2_25	GCTCCACCCTCCTCTGGGTTGAAAAGC	CAGGTGGGGATAAGAAACC
VH2_62	GCCTTGACTGAGAGGCATGGTCCTGAAATG	GCGGTGGCTCACGCCTGTAATCC
VH3_10B	CCGTCCTCCCTCTGCTGATGAAAACCAGC	CCCTGGGGAAATTTGACATGAGG

VH3_24	GGACCCACCATGGAGTTGGGACTGAGCTGGGTTTTCC	CGTTCCCTGGGGAAATTTGAC
VH3_27	CCAGGACGCTCTCATCTGCTCTGGTTCC	GTCCCACATCCTGACAGGAAATCAGC
VH3_29	CCAGGACACTCTCATCTACTCTGTGCACAGCCTTC	GCCTCCGGCAGCTGAGAAAGGAAACC
VH3_30	CTCCTGCAAGGCACAGTCACCTTATCTGG	CACAGCCAAGAATGCTGGTGTTTTGC
VH3_31	GGGCCCTCCTTCTACTGATGAAAACCAACCC	CTCCCTCCTTTCTTGCCTGCAGTGAGG
VH3_33	CTCCTGCGGGGCCTGTCATTTTATCTGG	GCCCTTGCACCACCTGCACTTGC
VH3_41	CCGGGACACTCTCCTCTGCTCTGA	GTGCACCGGCTTCCGGGTTGAC
VH3_42	CTCCTGCAAGGCACAGTCACCTTATCTGG	CTGCACCTGCTCCTGGGAC
VH3_44	GGGCCCTCCCTCTGCTGATGAAAACC	CCCCACGTTCTTGCAGGGAGGTTTGTG
VH3_45	CTGGGAGCCCCAGCCCTAGAATTCC	CGAGGCCCTCTGGGGAACTGTTAG
VH3_47	GGACACTCTCATCCGCTCTGGACACTGCCTTC	CCCGTTCCCGGCAGCTGAGAAAGG
VH3_48	CAGGCCTCTCACCCCAGAGCTTGCTAAATAG	CCTCTATAGCACCGGCCTCTGGGTTG
VH3_49	GAGAGGAGCCCTAGCCTGGGATTCC	CGCCCCTGTAGGAAGGTTTGTTTCTGC
VH3_50	GAGAGGAGGCCCAGACCTGGCATTTTCAGG	GTCCCACATCCTGACAGGAAATCAGC
VH3_51	GGTGTCCCACCCCAGAACTTGCCATATAGTAGG	GGCTGACTCTGATCAGTGGCTCCTGAG
VH3_52	CCCCAGGACACTCCTCATGCTCTTAGC	CCTGAGCAGCCCCTGCAGCTGATTTC
VH3_54	CCAGCCTGGGATTCCCAGGAGTTTCC	CGCCCCTGCAGGGAGGTTTG
VH3_55	GCGTCTCACCCCAGAGCTTGCTGTATAGTAGG	CCCTGAGTGTTCCTGCAGGGAGGTTTG
IGHVH3-21_human	GGTGATCAGGGCAAAGTGTTTATCACAGC	CTGGAGAAGTTCCCTGGGGAAATTTGA

Supplementary Table 2.

Effect of error rate on IgDiscover.
In order to get an impression of how sequencing error rate affects the discovery process, we created multiple versions of the H11M dataset (1380851 paired-end reads) by introducing additional substitution errors into the reads at rates between 0.1% and 3.0%. Results after running IgDiscover for three iterations are shown in Tab. X. An increase in error rate decreases the number of merged sequences and the number of IgBLAST-assigned sequences passing quality criteria to some degree, but the size of the final database decreases much more rapidly. This is to be expected since IgDiscover's germline filter relies on the presence of a minimum number of error-free copies of each V gene.
While this experiment simulates sequencing error rates being higher than a baseline, we observe that the size of the database seems to plateau between 0.0% and 0.5%. By extrapolation towards lower sequencing error rates, we hypothesize that only a small number of extra V genes would be detected in this case.

Extra substitutions	Merged sequences	IgBLAST assignments passing quality filtering	Size of final database
0.0%	1257467	856946	68
0.1%	1257172	863010	69
0.2%	1256929	861887	68
0.5%	1256115	838662	65
1.0%	1254533	777919	58
2.0%	1251246	659878	42
3.0%	1247623	560203	6
